# Draft genome of the oomycete pathogen *Phytophthora cactorum* strain LV007 isolated from European beech (*Fagus sylvatica*)

**DOI:** 10.1016/j.gdata.2017.05.010

**Published:** 2017-05-12

**Authors:** Laura J. Grenville-Briggs, Sandeep K. Kushwaha, Michelle R. Cleary, Johanna Witzell, Eugene I. Savenkov, Stephen C. Whisson, Aakash Chawade, Ramesh R. Vetukuri

**Affiliations:** a Department of Plant Protection biology, Swedish University of Agricultural Sciences, Alnarp, Sweden; b Department of Plant Breeding, Swedish University of Agricultural Sciences, Alnarp, Sweden; c National Bioinformatics Infrastructure Sweden (NBIS), Department of Biology, Lund University, Lund, Sweden; d Southern Swedish Forest Research Centre, Swedish University of Agricultural Sciences, Alnarp, Sweden; e Department of Plant Biology, Uppsala BioCenter, Linnean Centre of Plant Biology, Swedish University of Agricultural Sciences, Uppsala, Sweden; f Cell and Molecular Sciences, The James Hutton Institute, Invergowrie, Dundee, United Kingdom

## Abstract

*Phytophthora cactorum* is a broad host range phytopathogenic oomycete. *P. cactorum* strain LV007 was isolated from a diseased European Beech (*Fagus sylvatica*) in Malmö, Sweden in 2016. The draft genome of *P. cactorum* strain LV007 is 67.81 Mb. It contains 15,567 contigs and 21,876 predicted protein-coding genes. As reported for other phytopathogenic Phytophthora species, cytoplasmic effector proteins including RxLR and CRN families were identified. The genome sequence has been deposited at DDBJ/ENA/GenBank under the accession NBIJ00000000. The version described in this paper is version NBIJ01000000.

**Table:** 

Specifications	
Organism/cell line/tissue	*Phytophthora cactorum strain LV007*
Sex	Not applicable
Sequencer or array type	Illumina Hiseq2000
Data format	Assembled
Experimental factors	*P. cactorum* was isolated from a diseased European Beech (*Fagus sylvatica*) in Malmö, Sweden in 2016
Experimental features	Whole genome shotgun sequencing followed by genome assembly and gene description
Consent	Not applicable
Sample source location	Malmö, Sweden

## 1 Direct link to deposited data

https://www.ncbi.nlm.nih.gov/nuccore/NBIJ00000000.1

## 2 Introduction

*Phytophthora cactorum* is a plant pathogen with a diverse host range, infecting over 200 plant species [1]. These include trees, such as apple, pear and beech, as well as important horticultural crops such as strawberry [1], [2], [3]. Common symptoms of *P. cactorum* infection include crown, collar, fruit and root rot as well as foliar infections depending on the hosts it infects. Phylogenetically, *P. cactorum* is placed in clade 1 of the Phytophthora genus, which also includes the raspberry root rot pathogen *Phytophthora idaei* and *Phytophthora infestans* that causes late blight of potato and tomato [4]. In Europe, *P. cactorum* strains have been found to contribute to the increasing decline of ecologically valuable beech forests [5]. In southern Sweden, *P. cactorum* has been recognized as an increasing threat to urban and production forests, especially to broadleaved hosts [3]. Currently there are no means of chemical control that offer efficient control of *P. cactorum*. Thus, there is an urgent need to develop alternative measures for disease control that can mitigate the risk of disease spread. Relatively few tree-infecting oomycetes have been studied in detail at the genomic level so far [6]. However, draft genome sequences of several tree infecting Phytophthora species have recently been announced [7], [8], [9], [10]. To our knowledge, this is the first report of the genome sequence of a *P. cactorum* strain. Since our data is derived from a field strain, causing disease under natural conditions, the genome sequence may yield insights into the disease mechanisms employed by this species and thus facilitate the development of control strategies against *P. cactorum*.

## 3 Experimental design, materials and methods

*P. cactorum* was isolated from a diseased European Beech (*Fagus sylvatica*) in Malmö, Sweden in 2016. *P. cactorum* strain LV007 was cultured and DNA extracted as described [11], [12]. Paired-end (36.22 M reads) libraries were sequenced using the Illumina Hiseq2000 sequencing platform at MR DNA Molecular Research laboratory, USA. FastQC tools were used for raw data assessment [13]. Seqman NGEN v12 (DNASTAR) was used for *de novo* genome assembly. A total of 67.81 Mbs were assembled into 15,567 contigs (mean coverage, 133 ×). QUAST [14] was used to assess assembly quality (N50: 5788 bps; N75: 3102 bps; L50: 3034; and L75: 7067; Longest contig: 140,075 bps; Number of contigs > 25 kb: 138; Number of contigs > 10 kb: 1277). The assembly of *P. cactorum* was evaluated by BUSCO for completeness [15] based on a set of 1438 common fungal genes as benchmark universal single-copy orthologs (BUSCOs). 805 complete genes, 201 fragmented and 432 missing BUSCOs were found in this draft assembled genome sequence for this strain. The gene predictor Geneid (http://genome.crg.es/software/geneid/) predicted 21,876 genes using the previously sequenced plant pathogenic relative, *P. infestans* as a training model.

## 4 Data description

Primary annotation of predicted sequences revealed 9989 sequences having pfam domains. SignalP analysis predicted 1516 secretory proteins of which 257 are catalogued as RxLR class effector proteins and 15 proteins grouped as predicted crinkler (CRN) class effectors. CAZy proteins have been explored as described [16], [17]: Glycoside hydrolases (603), glycosyltransferases (766), carbohydrate binding modules (596), polysaccharide lyases (120), and carbohydrate esterases (71). Redox enzymes (56) were also identified.

## Conflict of interest statement

The authors declare that they have no conflict of interest.
